# The Curcumin Analog GO-Y030 Controls the Generation and Stability of Regulatory T Cells

**DOI:** 10.3389/fimmu.2021.687669

**Published:** 2021-06-23

**Authors:** Takashi MaruYama, Shuhei Kobayashi, Hiroko Nakatsukasa, Yuki Moritoki, Daiki Taguchi, Yoichi Sunagawa, Tatsuya Morimoto, Atsuko Asao, Wenwen Jin, Yuji Owada, Naoto Ishii, Yoshiharu Iwabuchi, Akihiko Yoshimura, WanJun Chen, Hiroyuki Shibata

**Affiliations:** ^1^ Mucosal Immunology Section, National Institute of Dental and Craniofacial Research (NIDCR), National Institute of Health, Bethesda, MS, United States; ^2^ Department of Immunology, Graduate School of Medicine, Akita University, Akita, Japan; ^3^ Department of Organ Anatomy, Tohoku University Graduate School of Medicine, Miyagi, Japan; ^4^ Department of Microbiology and Immunology, Keio University School of Medicine, Tokyo, Japan; ^5^ Department of General Internal Medicine and Clinical Laboratory Medicine, Akita University Graduate School of Medicine, Akita, Japan; ^6^ Department of Clinical Oncology, Graduate School of Medicine, Akita University, Akita, Japan; ^7^ Division of Molecular Medicine, School of Pharmaceutical Sciences, University of Shizuoka, Shizuoka, Japan; ^8^ Department of Microbiology and Immunology, Graduate School of Medicine, Tohoku University, Miyagi, Japan; ^9^ Department of Organic Chemistry, Graduate School of Pharmaceutics, Tohoku University, Miyagi, Japan

**Keywords:** Foxp3, GO-Y030, TGF-beta 1, gene regulating, regulatory T cell

## Abstract

Regulatory T cells (Tregs) play a crucial role in preventing antitumor immune responses in cancer tissues. Cancer tissues produce large amounts of transforming growth factor beta (TGF-β), which promotes the generation of Foxp3^+^ Tregs from naïve CD4^+^ T cells in the local tumor microenvironment. TGF-β activates nuclear factor kappa B (NF-κB)/p300 and SMAD signaling, which increases the number of acetylated histones at the *Foxp3* locus and induces *Foxp3* gene expression. TGF-β also helps stabilize Foxp3 expression. The curcumin analog and antitumor agent, GO-Y030, prevented the TGF-β-induced generation of Tregs by preventing p300 from accelerating NF-κB-induced Foxp3 expression. Moreover, the addition of GO-Y030 resulted in a significant reduction in the number of acetylated histones at the Foxp3 promoter and at the conserved noncoding sequence 1 regions that are generated in response to TGF-β. *In vivo* tumor models demonstrated that GO-Y030-treatment prevented tumor growth and reduced the Foxp3^+^ Tregs population in tumor-infiltrating lymphocytes. Therefore, GO-Y030 exerts a potent anticancer effect by controlling Treg generation and stability.

## Introduction

Transforming growth factor beta (TGF-β) is a cytokine with multiple functions related to cancer, including its potential to promote metastasis, angiogenesis, and fibroblast activation (1). TGF-β is secreted by several types of cancer cells (2–4); moreover, the autocrine TGF-β pathway mediates cancer initiation and progression. TGF-β also plays a crucial role in maintaining immune homeostasis *via* regulating the generation and function of regulatory T cells (Tregs) (5, 6). TGF-β can induce the generation of peripheral Tregs, primarily in the intestine (7). Mechanistically, the TGF-β-induced activation of SMAD2/3 enriches the conserved noncoding sequence 1 (CNS1) of Foxp3 and promotes *Foxp3* gene expression in cooperation with numerous additional transcription factors, including AP-1, a nuclear factor of activated T cells, and nuclear factor kappa B (NF-κB) (8, 9). Tregs also produce TGF-β and thereby limit the differentiation of CD8^+^ and CD4^+^ into cytolytic and Th1 cells, respectively (10). In the tumor microenvironment, Tregs suppress the cytotoxic responses of tumor-specific CD8^+^ T cells (11). Similarly, Chen et al. (12) reported that the TGF-β receptor on CD8^+^ T cells plays a crucial role in modulating Treg-dependent antitumor immunity (12). Consequently, the therapeutic potential of TGF-β blockers for cancer immunotherapy has been evaluated (13, 14).

Curcumin is a major component of turmeric (*Curcuma longa*) that can induce apoptosis in many types of cancer cells (15–17). Clinical trials have revealed that the oral administration of curcumin (3.6 g/kg, daily) had positive therapeutic effects in a variety of cancer patients (18–20). Curcumin also inhibits the generation and stability of Tregs in the tumor microenvironment (21, 22) and enhances the antitumor impact of immune checkpoint inhibitors (23). However, high doses of curcumin are necessary to achieve measurable anticancer effects, and the molecular mechanisms by which curcumin controls the generation and stability of Tregs remain unclear.

To address these concerns, we synthesized the curcumin analog, GO-Y030 (24). We reported that GO-Y030 administration suppressed cancer cell growth to a greater extent than that observed in response to curcumin alone in both *in vitro* and *in vivo* studies (24, 25). The 50% growth inhibitory concentration for GO-Y030 targeting the human gastric tumor cell line was shown to be 20-fold lower than that determined for underivatized curcumin (26). Here we found that GO-Y030 inhibited the generation, stability, and suppressive function of Tregs more efficiently than did curcumin. Mechanistically, GO-Y030 inhibited p300-enhanced NF-κB-induced Foxp3 expression. As such, our findings suggest that GO-Y030 has dual antitumor activity.

## Materials and Methods

### Experimental Models

All experiments in this study were performed according to the guidelines approved by the Institutional Animal Care and Use Committee of Akita University, Akita, Japan, Keio University, Tokyo, Japan and the National Institute of Dental and Craniofacial Research (NIDCR), Bethesda, MD, USA. All methodologies were performed in accordance with the relevant guidelines and regulations of Akita University and the NIDCR.

### Mice

C57BL/6 (CD45.2) mice were purchased from CLEA Japan, Inc. (Tokyo, Japan) and from Jackson Laboratory (Bar Harbor, ME, USA). C57BL/6 congenic CD45.1 mice and Foxp3-GFP mice were purchased from Jackson Laboratory. Mice aged 7–12 weeks old were used in this study and were maintained in specific pathogen-free conditions at the animal facilities in the Akita University and the NIDCR.

### Enzyme-Linked Immunosorbent Assay (ELISA)

SMAD3 (pSer423/S425) was quantitatively evaluated in T cell extracts using the SMAD3 (pSer423/S425) ELISA kit (Abcam, Cambridge, UK) according to the manufacturer’s instructions. HAT activity in cultured naïve CD4^+^ T cells was measured using the EpiQuick™ HAT activity/inhibition assay kit (EpiGentek, Farmingdale, NY) according to the manufacturer’s instruction. The absorbance was read at 450 nm in a Multiskan Fc Type 357 plate reader (Thermo Fisher Scientific, Waltham, MA, USA). ELISA kits for TGF-β1 (DuoSet^®^, R&D systems, Inc., Minneapolis, MN) and IL-10 (eBioscience, San Diego, CA) were used to quantify respective cytokines in the culture supernatants according to the manufacturers’ protocols.

### Flow Cytometry

Intranuclear FOXP3 staining was performed on cells that were fixed and permeabilized using the FOXP3 Staining Buffer Kit (eBioscience, San Diego, CA, USA) according to the manufacturer’s instructions. These cells were stained for 30 min at 4°C in the dark with anti-Foxp3 (FJK16s). Zombie Yellow™ Fixable Viability Kit (BioLegend, San Diego, CA, USA) staining was used to identify dead cells, according to the manufacturer’s instructions. Dead cells were also identified with DAPI (Thermo Fisher Scientific, Waltham, MA, USA) or FITC-conjugated Annexin V (eBioscience) and propidium iodide (eBioscience) according to the manufacturer’s instructions. The cells were analyzed by flow cytometry using a BD FACS Aria™ III (BD Bioscience, San Jose, CA), BD FACSymphony™ (BD Bioscience), Canto II (BD Bioscience), or Cytomics FC500 (Beckman Coulter, Brea, CA, USA). The data were analyzed using FlowJo software, Tree-star version (Ashland, OR, USA).

### Antibodies

FITC- or Pacific Blue-conjugated anti-mouse CD4 (GK1.5), Brilliant Violet 510™-conjugated anti-mouse CD4 (MR4-5), APC-conjugated anti-mouse CD45.1 (A20), FITC-conjugated anti-mouse TNF-α (MP6-XT22), APC-conjugated anti-mouse CTLA4 (UC10-4B9), and APC-conjugated anti-mouse GITR (DTA-1) antibodies were purchased from Biolegend. PE-conjugated, APC-conjugated, or Pacific Blue-conjugated anti-mouse Foxp3 antibodies (FJK-16S); PerCP-Cy5.5-conjugated anti-mouse CD45.1 (A20), PE-conjugated anti-mouse PD-1(J43), FITC-conjugated anti-mouse Ki67 (SolA15), APC-conjugated anti-mouse IFN-γ (XMG1.2), anti-IFN-γ (R4-6A2), and anti-IL-4 (11B11) antibodies were purchased from eBioscience. Alexa Fluor 647 Mouse anti-STAT5 (pY694) and PE-conjugated anti-STAT3 (pY705) antibodies were purchased from BD bioscience. Anti-Phospho SMAD3 (phosphor S423+425′ EP823Y) and anti-SMAD3 (AF9F7) antibodies were purchased from Abcam. Anti-GAPDH (14C10) antibodies were purchased from Cell Signaling Technology (Danvers, MA, USA).

### T Cell Cultures

The mouse CD4^+^CD62L^hi^ T Cell Isolation Kit was used to isolate naïve CD4^+^ T cells from mouse spleens according to the manufacturer’s instructions (Miltenyi Biotec, Bergisch Gladbach, Germany). Splenic CD4^+^CD25^+^ Tregs were isolated using the mouse CD4^+^CD25^+^ T Cell Isolation Kit according to the manufacturer’s instructions (Miltenyi Biotec). Purified cells (0.5 × 10^6^ cells/ml) were cultured at 37°C in RPMI-1640 containing 10% fetal calf serum, penicillin/streptomycin, and 50 μM 2-mercaptoethanol with 1 μg/ml plate-bound anti-CD3 (eBioscience) and 1 μg/ml soluble anti-CD28 (eBioscience) for 18 h to 3 days, as indicated in each experiment. For Treg differentiation, 2 ng/ml recombinant human TGF-β1 (Peprotech, Rocky Hill, NJ) was added to the cultures. Purified human naïve CD4^+^ T cells (0.4 × 10^6^ cells/ml) were cultured at 37°C in X-VIVO 15 using Dynabeads™ Human T-activator CD3/CD28 (Thermo Fisher Scientific) for 3 days. For human Treg differentiation, 2 ng/ml recombinant human TGF-β1 (Peprotech) was added to the cultures.

### Chromatin Immunoprecipitation (ChIP) Assay

Naïve CD4^+^ T cells were activated in culture with plate-bound anti-CD3 (1 μg/ml) and soluble anti-CD28 (1 μg/ml) for 3 days together with human TGF-β1 (2 ng/ml) in the presence or absence of curcumin (1 μM) or GO-Y030 (0.1 μM). ChIP was performed with anti-acetyl histone H3 (clone K27) (Cell Signaling), anti-p300/CREB binding protein (CBP; Cell Signaling), and normal rabbit IgG (Cell Signaling) as previously described (27, 28). Input and immunoprecipitated DNAs were analyzed with SYBR Premix EX Taq (Takara Bio, Shiga, Japan) by quantitative polymerase chain reaction (qPCR) using a LightCycler II (Roche, Basel, Switzerland). The following primer pairs were used for the qPCR experiments: for the Foxp3 promoter, 5′-TTCCTCCCGCTCTCTGACTCT-3′ and 5′-AAGCGCCAGTTGTGTACAAATATC-3′; and for the Foxp3 CNS1, 5′-GTTTTGTGTTTTAAGTCTTTTGCACTTG-3′ and 5′-CAGTAAATGGAAAAAATGAAGCCATA-3′.

### Reporter Assay

HEK293 cells were transfected with the pGL4-mouse Foxp3 promoter [−1702 to +174; (29)], pcDNA3, pcDNA3-FLAG-tagged mouse NF-kB subunit p65 (30), and pcDNA3-HA-tagged human p300 (31) using the calcium phosphate–DNA coprecipitation method (30). Luciferase activity was measured with the Dual-Luciferase Reporter Assay System (Promega) or the Duo-Luciferase Assay Kit (Genecopoeia, Rockville, MD, USA) according to the manufacturers’ instructions.

### Real-Time PCR

Total RNA was collected using the RNeasy Mini Kit (Qiagen, Venlo, the Netherlands), followed by cDNA synthesis with the PrimeScript II 1^st^ Strand cDNA Synthesis Kit (Takara Bio, Shiga, Japan). The resulting cDNA was evaluated by qPCR using an Applied Biosystems 7500 real-time PCR system (Thermo Fisher Scientific) or QuantStudio3 (Thermo Fisher Scientific) instrument and SYBR Premix EX Taq (Takara Bio) or TaqMan™ Gene Expression Master Mix (Thermo Fisher Scientific). The primer pairs used for the qPCR experiments are shown in **Table S1**.

### Bisulfate Sequencing

Genomic DNA was isolated from cultured CD4^+^CD25^+^ Tregs and evaluated by modified bisulfite sequencing with the MethylEasy Xceed DNA Modification Kit (Human Genetic Signatures, Randwick, Australia). The methods for amplifying and TA cloning of the Foxp3–CNS2 region were as previously described (32). Sequence analyses were performed by Eurofins Genomics (Tokyo, Japan).

### Treg Suppression Assay

CD4^+^CD25^−^ and CD4^+^CD25^+^ T cells were isolated using the mouse CD4^+^CD25^+^ T Cell Isolation Kit according to the manufacturer’s instructions (Miltenyi Biotec, Bergisch Gladbach, Germany). CD8^+^ T cells were isolated using the mouse CD8^+^ T Cell Isolation Kit according to the manufacturer’s instructions (Miltenyi Biotec, Bergisch Gladbach, Germany). For the CD4^+^CD25^+^ Treg suppression assay, CD4^+^CD25^+^ T cells were expanded in the presence of 10 ng/ml hIL-2 and 10 μg/ml anti-IFN-γ with or without 0.25 μM GO-Y030 for 3 days. Carboxyfluorescein succinimidyl ester (CFSE; Dojindo)- or CellTrace™ Violet Dye (Thermo Fisher Scientific)-labeled CD4^+^CD25^−^ T cells (1 × 10^5^ cells) isolated from CD45.1 mice were cultured in a 96-well plate with Dynabeads™ T-activator CD3/CD28 (Veritask, Tokyo, Japan) in the presence or absence of CD4^+^CD25^+^ T cells.

### Cell Proliferation Assay

Naïve CD4^+^ T cells were cultured for 72 h, after which 1/10 volume of Cell Counting Kit 8 reagent (Dojindo, Osaka, Japan) was added to provide an assessment of lactate dehydrogenase activity in live cells. After 4 h of incubation at 37°C under 5% CO_2_, absorbances were read at 450 and 595 nm (background) using a Multiskan Fc Type 357 plate reader (Thermo Fisher Scientific). CD8^+^ T cells were co-cultured with DMSO- or GO-Y030-treated CD4^+^CD25^+^ Tregs in the presence of anti-CD3/28 with Dynabeads™ T-activator CD3/CD28 (Veritask) for 3 days, after which 1/10 volume of Cell Counting Kit 8 reagent (APExBio, Houston, TX) was added. After 2 h of incubation at 37°C under 5% CO_2_, the absorbance was read at 450 using a SpectraMax Plus 384 plate reader (Molecular Devices, San Jose, CA).

### Tumor Model

B16-F10 melanoma cells (2.5 × 10^5^ cells/100 μl of PBS) were subcutaneously injected into the side flank of the C57/BL6 mice (Day 0). Seven days after tumor cell injection, we started to measure the size of the tumors (length and wide) and intraperitoneally injected DMSO-PBS, curcumin-PBS (5 mg/kg), or GO-Y030-PBS (5 mg/kg). Tumor volume (mm^3^) was calculated using the following formula: 0.5 × length (mm) × width (mm) × width (mm). Thirteen to sixteen days after tumor cell injection, we harvested the tumors and washed them with pre-cooled PBS. Then, the tumors were cut using scissors and digested using 1.25 mg/ml collagenase IV (Roche, Rotkreuz, Switzerland) and 0.1 mg/ml DNase I (Sigma-Aldrich) in PBS at 37°C in a shaker for 1 h. After digestion, we added 15 ml of PBS to the tumors and washed the digested tumors by centrifugation (300 × g, 5 min, 4°C). To purify the tumor-infiltrating lymphocytes, we re-suspended the tumor digestion using a 40% Percoll gradient **(**GE Healthcare, Chicago, IL**)** and added an 80% Percoll gradient into the bottom, followed by centrifugation at 550 × g for 30 min at 4°C. Then, we collected the middle layers and immediately washed them with PBS.

### Statistical Analysis

All statistical analyses were performed using GraphPad Prism 5 software (GraphPad Software). Statistical significance was identified as *p* < 0.05 (* p < 0.05, ** p < 0.01, and *** p < 0.001).

## Results

### GO-Y030 Inhibits the Generation of Foxp3^+^ Tregs

TGF-β produced by cancer cells contributes to the generation of Tregs in the tumor microenvironment (33). Curcumin induces apoptosis in tumor cells and prevents Treg generation within the tumor microenvironment (21). TGF-β also prevents T cell death (34). We therefore examined whether the curcumin analog, GO-Y030, an agent characterized by antitumor activity, could inhibit the TGF-β-induced generation of Foxp3^+^ Tregs and promote T cell viability *in vitro*.

**Figure 1:**
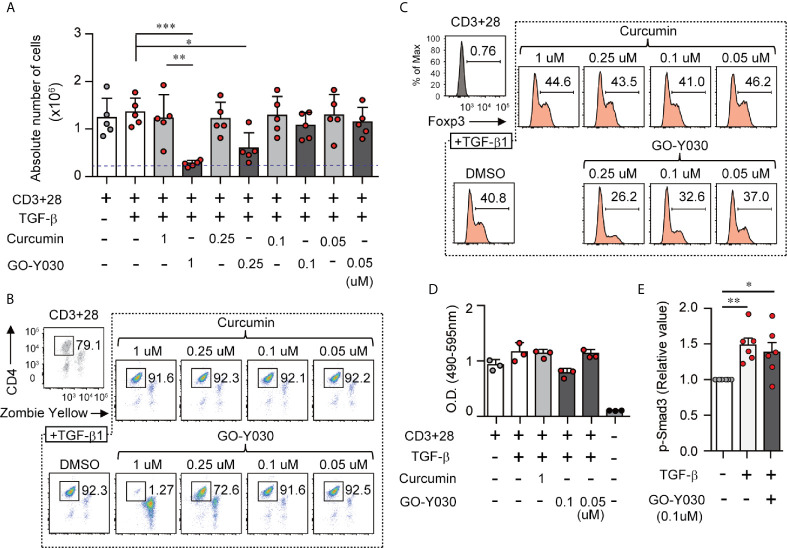
GO-Y030 inhibits TGF-β-induced Foxp3^+^ Tregs. **(A)** Cell counts. Naïve splenic CD4^+^ T cells were cultured with or without 2 ng/ml TGFβ and 1 µM curcumin or concentrations of GO-Y030 as indicated for a period of 3 days. The blue threshold shows the number of CD4^+^ T cells (0.25 × 10^6^ cells) before culturing (0 h). **(B, C)** Frequency of Foxp3^+^ Tregs in the total CD4^+^ cell population. Naïve splenic CD4^+^ T cells were cultured with or without 2 ng/ml TGFβ and 1 µM curcumin or concentrations of GO-Y030 as indicated for a period of 3 days. The data are representative of at least three independent experiments. **(D)** Relative live cell counts. Naïve splenic CD4^+^ T cells were cultured with or without 2 ng/ml TGFβ and concentrations of GO-Y030 as indicated for 72 h, followed by the addition of the cell counting reagent. The horizontal bars represent the mean and standard deviation. **(E)** The relative value of phosphorylation of SMAD3. Splenic naïve CD4^+^ T cells were cultured in the presence or absence of 2 ng/ml TGFβ or 0.1 µM GO-Y030 for 1 h. The absorbance at 450 nm in the absence of TGFβ is set as “1.” The horizontal bars represent the mean and standard deviation. The findings were evaluated with a one-way analysis of variance (ANOVA) with *post-hoc* Tukey’s multiple comparisons test. *P< 0.05, **P < 0.01, ***P < 0.001.

The experiments shown in **Figures 1A, B** and **Figures S1A, B** explore the impact of GO-Y030 on T cell viability. We found that the addition of 1 µM GO-Y030 resulted in T cell death response to TGF-β. GO-Y030 at a concentration of 0.25 µM had little impact on T cell viability compared with the results obtained from 0.25 µM curcumin or DMSO (diluent) alone. However, 0.1 µM GO-Y030 had a minimal impact on T cell death compared with the responses observed from 0.1 µM curcumin or DMSO alone. Annexin V and propidium iodide staining revealed that 1 µM GO-Y030 treatment on CD4^+^ T cells significantly induced apoptotic cell death even in the presence of TGF-β (**Figures S2A**,** B**). We also confirmed that GO-Y030 at concentrations of 0.01 to 0.25 µM had little or no impact on CD4^+^ T cell viability (**Figures S2A–C**). Conversely, CD8^+^ T cells had greater resistance against GO-Y030-induced apoptotic cell death than did CD4^+^ T cells (**Figures S2D–F**). We also found that 0.1 to 0.05 µM GO-Y030 treatment significantly diminished TGF-β-induced Foxp3^+^ Treg generation compared with the responses observed to curcumin or DMSO alone (**Figures S3A**-**B**). We also evaluated Foxp3 expression in CD4^+^ Zombie Yellow-negative populations (i.e., live CD4^+^ T cells). We found that GO-Y030 was a strong inhibitor of TGF-β-induced Foxp3^+^ Treg generation (**Figure 1C** and **Figure S4**). By contrast, the Cell Counting Kit-8 revealed that 0.1 µM GO-Y030 had little to no impact on T cell survival compared with responses to the DMSO diluent control (**Figure 1D**). In human naïve CD4^+^ T cells, 0.39 µM GO-Y030 can inhibit TGF-β-induced Foxp3^+^ Treg generation without inducing cell death (**Figures S5A–C**). These results indicate that GO-Y030 has more potent inhibitory activity with respect to Treg generation than does underivatized curcumin alone.

### GO-Y030 Inhibits Histone Acetyltransferase p300 Activity and Subsequently Foxp3 Gene Expression

Next, we focused on the molecular mechanisms of the GO-Y030-mediated inhibition of TGF-β-induced Treg generation. The representative TGF-β signal transducer, SMAD, plays a key role in Treg generation (35). TGF-β rapidly stimulates T cells to activate SMAD3 (9). Here we confirmed that TGF-β induced SMAD3 activation (**Figure 1E** and **Figures S6A, B**). The addition of GO-Y030 to the culture media had no impact on the TGF-β-induced activation of SMAD3 (**Figure 1E** and **Figures S6A, B**). Thus, we can conclude that the TGF-β/SMAD signaling pathway is not involved in inhibiting the TGF-β-induced generation of Tregs observed in response to GO-Y030.

**Figure 2:**
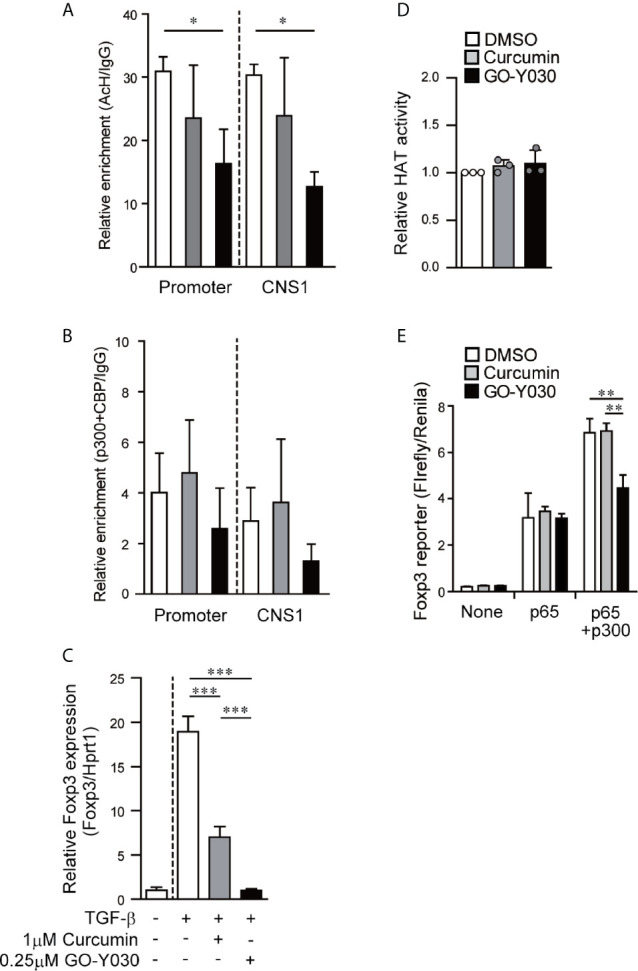
GO-Y030 reduces the level of histone acetylation at the Foxp3 locus and prevents Foxp3 gene expression. **(A, B)** Real-time PCR documenting the relative amount of chromatin immunoprecipitation [acetyl histone H3K27/IgG **(A)** and acetyl transferase p300+CBP/IgG **(B)**] at the Foxp3 locus (n = 3) using cultured naïve CD4+ T cells (24 h) in the presence of 2 ng/ml TGF-β with or without curcumin, GO-Y030, and DMSO. White bar: DMSO; gray bar: 1 µM curcumin; black bar: 0.1 µM GO-Y030. The horizontal bars represent the mean and standard deviation. The data are representative of at least three independent experiments. A one-way ANOVA with *post-hoc* Tukey’s multiple comparisons test was applied. **(C)** Naïve CD4+ T cells were incubated with 1 μM curcumin, 0.25 μM GO-Y030, or DMSO diluent control for 24 h. A representative value for Foxp3 expression in DMSO-treated naïve CD4+ T cells without TGF-β stimulation was set as “1.” The horizontal bars represent the mean and standard deviation. The data are representative of four independent experiments performed in triplicate. A one-way ANOVA with *post-hoc* Tukey’s multiple comparisons test was applied. **(D)** Naïve CD4+ T cells were incubated with 1 μM curcumin, 0.25 μM GO-Y030, or DMSO diluent control in the presence of TGF-β stimulation for 48 h. The data are pooled from three independent experiments. A representative value for HAT activity in DMSO-treated naïve CD4+ T cells with TGF-β stimulation was set as “1.” The horizontal bars represent the mean and standard deviation. **(E)** The Foxp3 promoter assay was performed in HEK293 cells. Twenty-four hours before plasmid transfection, the cells were incubated with 1 µM curcumin, 1 µM GO-Y030, or DMSO control. The data are representative of at least three independent experiments (n = 3); the mean and standard deviation are shown. A one-way ANOVA with *post-hoc* Tukey’s multiple comparisons test was applied. *P< 0.05, **P < 0.01, ***P < 0.001.

The Foxp3 promoter and CNS1 regions play critical roles in Treg generation (9, 29, 36). In these regions, the histone acetylation revealed an open chromatin conformation that was associated with *Foxp3* gene expression (35). Likewise, histone acetyltransferase p300-deficient T cells present with lower levels of TGF-β-induced acetyl histone H3 in the Foxp3 promoter/CNS1 regions and display lower levels of *Foxp3* gene expression (37). Our ChIP assay demonstrated that GO-Y030 treatment significantly reduced the amount of histone H3 acetylation at the Foxp3 promoter and the CNS1 regions compared with results obtained from the DMSO diluent control (**Figure 2A**). GO-Y030 treatment also resulted in diminished levels of p300/CBP at the Foxp3 promoter and the CNS1 regions (**Figure 2B**). At this point, GO-Y030 prevents *Foxp3* gene expression in response to TGF-β (**Figure 2C**). We evaluated HAT activity in cultured naïve CD4^+^ T cells in the presence of TGF-β; however, GO-Y030 did not show any affect against HAT activity (**Figure 2D**).

Since the overexpression of histone acetyltransferase p300 had little to no impact on Foxp3 promoter activity (**Figures S7A, B**), we focused instead on the mechanisms *via* which p300 interacts with NF-κB to enhance NF-κB-induced Foxp3 transcription (37, 38). We found that GO-Y030 had no impact on NF-κB-induced Foxp3 promoter activity (**Figure 2E**). Additionally, GO-Y030-treatment did not inhibit Foxp3 promoter activity in the presence of p300 (**Figures S7A****, B**). However, GO-Y030 did significantly reduce NF-κB-induced Foxp3 promoter activity in the presence of p300 compared with activity levels from the DMSO control (**Figure 2E**). These results suggest that GO-Y030 could inhibit the p300-enhanced NF-κB-induction of Foxp3 promoter activity and histone acetylation at the *Foxp3* gene.

### GO-Y030 Reduces Treg Stability

**Figure 3:**
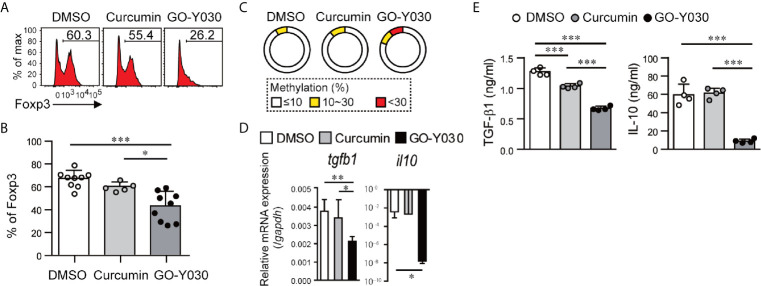
GO-Y030 reduces the stability of Foxp3 expression in CD4^+^CD25^+^ T cells. **(A)** Representative FACS plots of Foxp3^+^ cells. Splenic CD4^+^CD25^+^ T cells were stimulated with anti-CD3 (1 µg/ml) and anti-CD28 (1 µg/ml) in the presence or absence of 1 µM curcumin or 0.25 µM GO-Y030 for 18 h. **(B)** The horizontal bars represent the mean and standard deviation. The data were pooled from at least four independent experiments in **(A)**. **(C)** Bisulfate sequencing of Foxp3–CNS2 regions in cultured CD4^+^CD25^+^ T cells for 18 h using anti-CD3 (1 µg/ml) and anti-CD28 (1 µg/ml) in the presence or absence of 1 µM curcumin or 0.25 µM GO-Y030. The parentage of CpG methylation status in the Foxp3-CNS2 regions is shown in the sector graphs; 9–11 cDNA clones were sequenced from each subset. The data are representative of at least two independent experiments. The color of the graph indicates the percentage of methylation in the Foxp3-CNS2 locus: white, 10% or less; yellow, above 10% but less than or equal to 30%; red, above 30%. **(D)** Relative *tgfb1* and *il10* expression (n = 4–8, mean ± standard deviation) by real-time PCR. The data were normalized to relative *Gapdh* expression. **(E)** TGF-β1 production from cultured Tregs in the presence or absence of 1 µM curcumin or 0.25 µM GO-Y030 for 3 days. The graph shows the mean and standard deviation. A one-way ANOVA with *post-hoc* Tukey’s multiple comparisons test was applied **(D, E)**. *P< 0.05, **P < 0.01, ***P < 0.001.

We then evaluated the effect of GO-Y030 on Treg stability in *in vitro* experiments. We confirmed that approximately 85% of CD4^+^CD25^+^ T cells expressed Foxp3 (**Figure S8**). We found that CD4^+^CD25^+^ T cells treated with GO-Y030 for 18 h showed a significantly reduced expression of Foxp3 compared with cells incubated with the DMSO control (**Figures 3A, B** and **Figure S9A**). Moreover, CD4^+^CD25^+^ T cells treated with GO-Y030 showed a reduced mean fluorescence intensity for Foxp3 compared with cells responding to curcumin or DMSO alone (**Figure S9A**). We also confirmed that the survival rates of the cultured CD4^+^CD25^+^ T cells were comparable among the DMSO, curcumin, and GO-Y030 treatments (**Figure S9B**). A 5 µM curcumin concentration resulted in reduced Foxp3 expression in CD4^+^CD25^+^ T cells compared with DMSO alone (22); however, at a concentration of 1 µM, curcumin did not result in any change in Foxp3 expression in these cells (**Figures 3A, B**). GO-Y030 treatment resulted in significantly reduced Foxp3 expression in CD4^+^CD25^+^ T cells at a concentration at least four times lower than that required for curcumin. During the 18 h *in vitro* Treg culture, neither curcumin nor GO-Y030 had any impact on Treg survival (**Figures S9B, C**) or the absolute number of Tregs detected (**Figure S9D**). We also confirmed that CD4^+^Foxp3-GFP^+^ Tregs treated with GO-Y030 for 18 h showed significantly reduced expressions of Foxp3-GFP compared with cells incubated with DMSO (**Figure S9E**). Furthermore, the de-methylation status of CNS2, a factor that has been associated with the stability of Foxp3^+^ Tregs (31, 35), was reduced in the GO-Y030-treated Tregs compared with those treated with curcumin or DMSO alone (**Figure 3C**). As such, we can conclude that the addition of GO-Y030 results in reduced Treg stability.

### GO-Y030 Has the Potential to Affect the Suppressive Function of Tregs

Tregs produce and release suppressor cytokines, including TGF-β and IL-10, that regulate cancer immunity. We have shown that the addition of GO-Y030 resulted in significant reductions in *tgfb1* and *il10* expression in cultured CD4^+^CD25^+^ Tregs (**Figures 3D, E**). GITR is a cell surface receptor that is highly expressed on Tregs and plays a critical role in cancer immunity (39, 40). GO-Y030 treatment resulted in reduced GITR expression on Tregs (**Figure S9F**). The co-inhibitory receptor, CTLA4, is also highly expressed on Tregs and plays critical roles in cancer immunity (41). However, GO-Y030 had no impact on CTLA4 expression on Tregs (**Figure S9G**).

**Figure 4:**
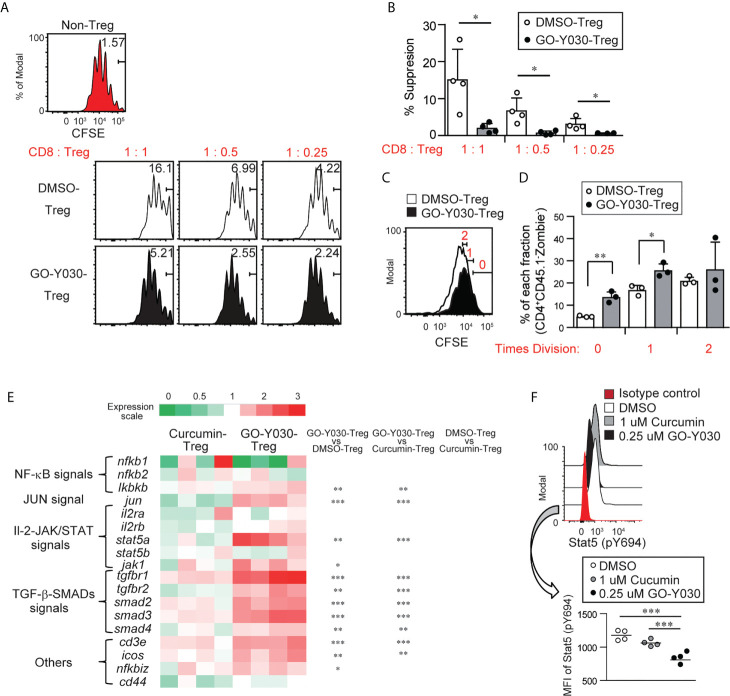
GO-Y030-mediated suppression of Tregs. **(A)** Proliferation ratio of CFSE-labeled CD8^+^ T cells isolated from CD45.1 mice and cultured with or without CD4^+^CD25^+^ Tregs for 72 h. The Tregs were treated with 0.25 µM GO-Y030 or DMSO control for 3 days before co-culturing. The CD8^+^CD45.1^+^ gated cell population is shown. **(B)** Relative suppressive of Tregs. The percentage of non-proliferation cells in non-Tregs is set to 0%. The data are representative of at least three independent experiments. The circles stand for independent experiments. The horizontal bars represent the mean and standard deviation. **(C, D)** The proliferation ratio of CFSE-labeled CD4^+^ CD25^+^ Tregs 3 days after the co-culture systems is shown. The ratio of CD8^+^ T cells to Tregs is 1:1. The circles stand for independent experiments. The graph shows the mean and standard deviation. **(E)** The heat map shows the real-time quantitative RT-PCR analysis results of DMSO-, 1 uM curcumin-, or 0.25 uM GO-Y030-treated CD4^+^ CD25^+^ Tregs for 3 days. The color scale is shown at the top of the heat map. Each genes’ expression in the DMSO-treated Tregs is as set as “1.” The data are from four independent experiments. **(F)** Stat5 (p694) expression in cultured CD4^+^CD25^+^ Tregs for 3 days with or without curcumin or GO-Y030. The data show the gated CD4^+^Zombie^-^ population. The graph shows the mean and standard deviation. The Student’s t-test **(B, D)** or one-way ANOVA with *post-hoc* Tukey’s multiple comparisons test **(E, F)** was used. *P< 0.05, **P < 0.01, ***P < 0.001.

The levels of Foxp3 in Tregs are associated with their suppressive ability (42). Because a high dose (~20 uM) of curcumin-treated CD4^+^CD25^+^ Tregs indicated the possibility of reducing suppressive abilities due to low Foxp3 expression (22), we evaluated the suppressive function of GO-Y030-treated Tregs *in vitro*. First, GO-Y030-treated CD4^+^CD25^+^ Tregs showed a significantly reduced suppression ability compared with DMSO-treated CD4^+^CD25^+^ Tregs (**Figures 4A, B**). Second, DMSO- or GO-Y030-treated CD4^+^CD25^+^ Tregs were labeled using CFSE; the results revealed that GO-Y030 treatment showed significantly reduced Treg proliferation in this co-culture system (**Figures 4C, D**). In the presence of IL-2 signaling, the Foxp3^+^ population was comparable between GO-Y030- and DMSO-treated CD4^+^CD25^+^ Tregs (**Figure S10A**). However, the Foxp3^+^ population from GO-Y030-treated CD4^+^CD25^+^ Tregs was reduced in this co-culture system (**Figure S10B**). Next, we focused on the IL-2/STAT5 signaling pathway, which plays important roles in both the stability of Foxp3 expression and the expansion of Foxp3^+^ Tregs even in the tumor microenvironment (35). Although GO-Y030-treated Tregs do not reduce STAT5 expression (**Figure 4E**), STAT5 phosphorylation was significantly reduced compared with that of DMSO- and curcumin-treated CD4^+^CD25^+^ Tregs (**Figure 4F**). Our real-time PCR (**Figure 4E** and **Figure S11A**) with Enrichr analyses (https://maayanlab.cloud/Enrichr/) also suggest that GO-Y030 controls IL-2/STAT5 signaling (Top-Ranked) in CD4^+^CD25^+^ Tregs (**Figures S11B, C**). One reason for this is that GO-Y030-treated CD4^+^CD25^+^ Tregs highly expressed SOCS1, a common γ chain inhibitor (43) (**Figure S11A**). SOCS1 expression in GO-Y030-treated Foxp3-GFP^+^ Tregs was also significantly higher than that in DMSO-treated Foxp3-GFP^+^ Tregs (**Figure S12A**). Additionally, we found that STAT5 activation, a signal molecule of the IL-2 receptor that plays a crucial role in the expansion of Tregs (35), was significantly reduced in cultured GO-Y030-treated CD4^+^CD25^+^ Tregs (**Figure 4F**). We also found that the expression of orphan receptor Rora, a Th17 driver, was higher in GO-Y030-treated Foxp3-GFP^+^ Tregs (**Figure S12A**) (44). Therefore, GO-Y030-treated Foxp3-GFP^+^ Tregs produced more IL-17A than did DMSO-treated Foxp3-GFP^+^ Tregs (**Figure S12B**). Overall, our results suggest that GO-Y030 has the potential to affect Tregs’ suppressive functions.

### GO-Y030 Controls Antitumor Immunity

**Figure 5:**
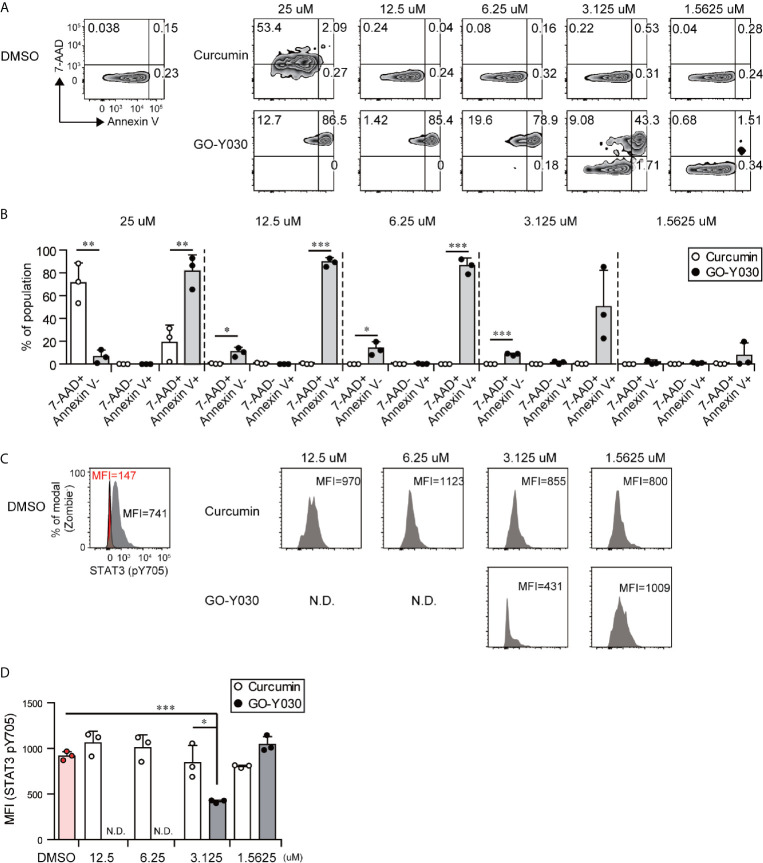
GO-Y030 induces tumor cell death and prevents STAT3 activation. **(A–D)** The B10-F16 tumor cell line was treated with each concentration of curcumin or GO-Y030 for 72 h. The data are representative at three independent experiments. **(A)** Representative 7-AAD and Annexin V staining by FACS. **(B)** Cell death percentages of each population. White, curcumin treatment; black, GO-Y030-treatment. **(C, D)** Representative Stat3 (pY705) mean fluorescent intensity by FACS analysis. Gray, isotype control; red, STAT3 (pY705). N.D., not detected live cells (Zombie^-^ population). A one-way ANOVA with *post-hoc* Tukey’s multiple comparison test was used. *P< 0.05, **P < 0.01, ***P < 0.001.

**Figure 6:**
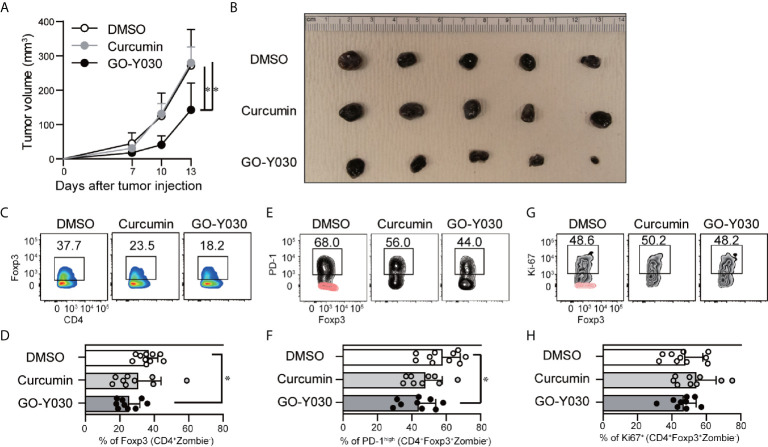
GO-Y030 reduced Foxp3^+^ Tregs in tumor-infiltrating lymphocytes. **(A)** Calculation of the tumor volume (mm^3^) for each day beginning 7 days after tumor injection (n = 9–10, mean + standard deviation). **(B)** Representative tumors in each group harvested at the end of the experiments as in **(A)**. **(C, D)** Representative intranuclear staining of Foxp3^+^ Tregs (CD4^+^Zombie^-^Foxp3^+^ population) in tumor-infiltrating lymphocytes. **(E, F)** Representative PD-1^high^ population of Tregs (CD4^+^Zombie^-^Foxp3^+^ population) in tumor-infiltrating lymphocytes. Red, isotype control; black, PD-1. **(G, H)** Representative Ki-67^+^ population in Tregs (CD4^+^Zombie^-^Foxp3^+^ population) in tumor-infiltrating lymphocytes. Red, isotype control; black, Ki-67. Data are representative of two independent experiments **(B, C, E, G)**. A one-way ANOVA with *post-hoc* Tukey’s multiple comparisons test was used **(A, D, F, H)**. The graph shows the mean and standard deviation. *P < 0.05.

GO-Y030 induces apoptosis in multiple tumor cells by inhibiting the activation of STAT3 (45). We confirmed that GO-Y030-treatment strongly reduced the survival of B16-F10 melanoma cells (**Figures 5A, B**) and STAT3 activation (**Figures 5C, D**) compared with curcumin treatment. Next, we addressed the role of GO-Y030 in tumor immunity *in vivo*. We found that 5 mg/kg GO-Y030 treatment significantly inhibited tumor growth *in vivo* (**Figures 6A, B**). Additionally, GO-Y030 treatment resulted in a significantly reduced Foxp3^+^ Tregs population in tumor-infiltrating lymphocytes compared with that from DMSO treatment (**Figures 6C, D**). PD-1^high^Foxp3^+^ Tregs play crucial roles in tumor immunity (46). Interestingly, GO-Y030-treated tumor models showed a decreased PD-1^high^Foxp3^+^ Tregs population in tumor-infiltrating lymphocytes (**Figures 6E, F**). We also evaluated Ki-67, a proliferation marker, in Foxp3^+^ Tregs; however, no significant differences were observed between DMSO, curcumin, and GO-Y030 treatment (**Figures 6G, H**). We also found that GO-Y030 did not prevent CD8α^+^ cells from infiltrating into the tumor side (**Figure S13A**). The CD8α^+^/Tregs ratio in tumors tended to be higher in GO-Y030 treated mice than in DMSO-treated mice (**Figure S13B**). GO-Y030 also did not prevent the proliferation of CD8^+^ cells in the tumor side (**Figures S13C, D**). Additionally, GO-Y030-treated tumor models tended to have more IFN-γ and TNF-α production from CD8α^+^ cells in tumor-infiltrating lymphocytes (**Figures S13E–H**). Thus, GO-Y030 has dual effects: it controls the generation and stability of Tregs and it plays a role in tumor cell death.

## Discussion

The curcumin analog GO-Y030 exerts its antitumor effects through a variety of mechanisms including inducing apoptosis, inhibiting IKKβ activation (25), blocking STAT3 activation (47), and inhibiting p300-HAT (48).

Tumor tissues produce large amounts of TGF-β, which plays a pivotal role in promoting the generation and stability of Foxp3^+^ Tregs in the local tumor microenvironment (21); this is a critical observation because Tregs localized in the tumor tissues can interfere with the antitumor immune response (49). Based on these findings, TGF-β and downstream signaling molecules may serve as novel targets for cancer immunotherapy (14, 50).

As a feature of the generation of Tregs, TGF-β signaling can quickly activate SMAD; this typically occurs within an hour of stimulation. Activated SMAD accumulates in the CNS1 regions of the *Foxp3* gene (9, 35). Subsequently, NF-κB accumulates at the promoter (51); p300 then cooperates with NF-κB to promote *Foxp3* expression. Our results revealed that the curcumin analog, GO-Y030, can inhibit p300-enhanced NF-κB-induced *Foxp3* expression. The CNS2 region of the *Foxp3* gene is also a key element involved in the stability of Tregs (31). P300 acts on this region to promote Treg stability (37); p300-HAT activity is likewise critical for suppressing the immune response initiated by Fox3^+^ Tregs (52). Moreover, TGF-β signaling plays a crucial role in promoting the accumulation of p300 on the CNS2 element of the *Foxp3* gene (36). CNS2 has an NF-κB binding element, but it is dispensable for *Foxp3* gene expression (53). Based on these observations, we proposed that GO-Y030 might reduce Treg stability by inhibiting the function of the NF-κB/p300 axis at the CNS2 region of the *Foxp3* gene. Furthermore, GO-Y030 inhibits the IL-2/STAT5 axis in Tregs, which plays important roles in the proliferation/stabilization of Tregs (54). Interestingly, SOCS1, a common γ chain inhibitor (43), showed significantly higher expression levels in GO-Y030-treated Tregs compared with DMSO- or curcumin-treated Tregs.

Curcumin was found to destabilize PD-L1 expression in cancer cells, which resulted in an increased number of tumor-infiltrating activated CD8^+^ T cells (55). Other studies have revealed that curcumin can suppress the interferon gamma (IFN-γ)-induced upregulation of PD-L1 expression in cancer cells (56). Moreover, studies in numerous animal models have shown that combination therapy with curcumin and the immune checkpoint inhibitor, anti-CTLA-4, significantly suppressed tumor growth.

Curcumin prevented the tumor-induced infiltration of Foxp3^+^ Tregs (57) and reduced the stability of Tregs (22). Interestingly, colorectal cancers characterized by abundant infiltration with highly stable Foxp3^(hi)^ Tregs were associated with a worse prognosis than those with low-stability Tregs (58). The administration of comparatively high doses of curcumin (5 µM *in vitro* and 50 mg/kg *in vivo*) resulted in both direct antitumor effects and actions at immune checkpoints *via* the inhibition of Treg generation and stability. By contrast, GO-Y030 at concentrations of 0.25 µM or less could sufficiently inhibit the generation of Tregs and reduce their stability.

Curcumin is also known to exert anti-inflammatory effects *via* the inhibition of cyclooxygenase-2, lipoxygenase, and inducible nitric oxide synthase (59). Under certain conditions, chronic inflammation can lead to tumor initiation (60); as such, curcumin may serve in a cancer-preventive mode in these settings (61). Curcumin’s anti-inflammatory effect is largely provided by its bis-(arylmethylidene) acetone structure (59). GO-Y030 maintains the bis-(arylmethylidene) acetone moiety and also exerts strong anti-inflammatory and cancer-preventive effects (62).

In summary, our findings reveal that the curcumin analog, GO-Y030, has potent antitumor effects and thus may be developed into a potential novel anticancer immunotherapeutic agent.

## Data Availability Statement

The raw data supporting the conclusions of this article will be made available by the authors, without undue reservation.

## Ethics Statement

All experiments in this study were performed according to the guidelines approved by the Institutional Animal Care and Use Committee of Akita University, Akita, Japan, Tohoku University, Miyagi, Japan and the National Institute of Dental and Craniofacial Research (NIDCR), Bethesda, MD, USA. All methodologies were performed in accordance with the relevant guidelines and regulations of Akita University, Tohoku University and NIDCR. Written informed consent for participation was not required for this study in accordance with the national legislation and the institutional requirements.

## Author Contributions

TMa conceived of and directed this study, designed and performed most of the experiments, analyzed the data, and wrote this manuscript. SK, HN, DT, and YS performed the experiments and analyzed the data. AA and WJ helped to perform the experiments and analyze the data. YM, YI, TMo, OY, and AY provided critical materials. NI and WC provided critical suggestions and materials. HS supervised the experiments and contributed to the editing of this manuscript. All authors contributed to the article and approved the submitted version.

## Funding

This research was supported in part by the Fund for the Promotion of Joint International Research [Fostering Joint International Research (B)] (18KK0257) to TMa, the Intramural Research Program of NIDCR to WC, and a Grant-in-Aid for Scientific Research (C) (20K11643) to HS.

## Conflict of Interest

The authors declare that the research was conducted in the absence of any commercial or financial relationships that could be construed as a potential conflict of interest.
