# Supplementary material for: The Curcumin Analog GO-Y030 Controls the Generation and Stability of Regulatory T Cells
Source: Front Immunol. 2021 Jun 23;12:687669. doi: 10.3389/fimmu.2021.687669 (PMC8261301; doi:10.3389/fimmu.2021.687669)
Supplement: Supplementary file 1 [file DataSheet_1.pdf]

Interleukin-10	Forward	5′-CCCATTCCTCGTCACGATCTC-3′
	Reverse	5′-TCAGACTGGTTTGGGATAGGTTT-3′
TGF-β1	Forward	5′-CTCCCGTGGCTTCTAGTGC-3′
	Reverse	5′-GCCTTAGTTTGGACAGGATCTG-3′
GAPDH	Forward	5′-CCAGGTTGTCTCCTGCGACTT-3′
	Reverse	5′-CCTGTTGCTGTAGCCGTATTCA-3′
Foxp3-promoter	Forward	5′-TTCCTCCCGCTCTCTGACTCT-3
	Reverse	5′-AAGCGCCAGTTGTGTACAAATATC-3′
Foxp3-CNS1	Forward	5′-GTTTTGTGTTTTAAGTCTTTTGCACTTG-3′
	Reverse	5′-CAGTAAATGGAAAAAATGAAGCCATA-3′
**(For Taqman)**		**Cat#**
Hprt1		Mm00446968_m1
Foxp3		Mm00475162_m1
Tgfb1		Mm00441724_m1
Il10		Mm01288386_m1
Tnf		Mm00443258_m1
Tgfbr1		Mm00436964_m1
Tgfbr2		Mm03024091_m1
Smad6		Mm00484738_m1
Smad7		Mm00484742_m1
Rel		Mm01239661_m1
Tbx1		Mm00448949_m1
Rorc		Mm01261022_m1
Nfkb1		Mm00446968_m1
Nfkb2		Mm00476361_m1
Ikbkb		Mm01222247_m1
Jun		Mm00495062_s1
Il2ra		Mm01340213_m1
Il2rb		Mm00434268_m1
Smad2		Mm00487530_m1
Smad3		Mm00489637_m1
Smad4		Mm03023996_m1
Cd3e		Mm01179194_m1
Icos		Mm00497600_m1
Nfkbiz		Mm00600522_m1
Cd44		Mm01277161_m1
Stat5a		Mm03053818_s1
Stat5b		Mm0083989_m1
Jak1		Mm00600614_m1
Hdac4		Mm01299552_m1
Ifng		Mm01168134_m1
Il2		Mm00434256_m1
Il5		Mm00439646_m1
Il13		Mm00434204_m1
Inha		Mm00439683_m1
Rorc		Mm01261022_m1
Batf		Mm00479410_m1
Stat3		Mm00456961_m1

Myd88	Mm00440338_m1
Myc	Mm00487803_m1
Hk1	Mm00439344_m1
Hk2	Mm00443385_m1
Socs1	Mm00782550_s1
Socs3	Mm00545913_s1
Slc2a1	Mm00441480_m1
Slc2a3	Mm00441483_m1
Runx1	Mm01213404_m1
Runx3	Mm00490666_m1
Tcf12	Mm00441699_m1
Cd274	Mm03048248_m1
Rara	Mm01296312_m1
Rora	Mm01173766_m1

Supplementary Table 1: Primers List

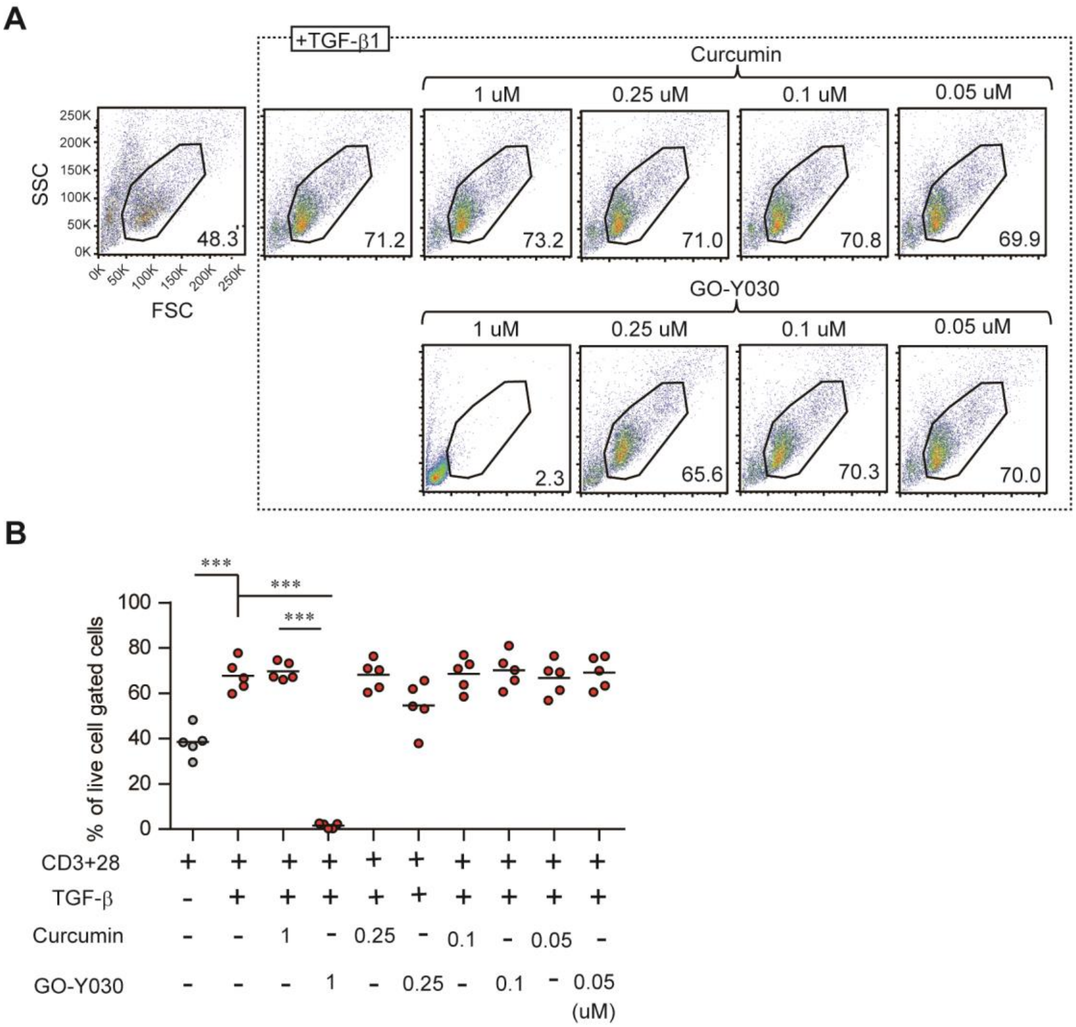

Supplementary Figure 1: Naive CD4⁺ T cells from C57/BL6 mice cultured in each concentration of GO-Y-030.

(**A**, **B**) Representative SSC and FSC FACS analysis at five independent experiments. Purified naïve CD4⁺ T cells cultured for three days, and then gated live cell population according to SSC and FSC. Statistical analyses were performed in each concentration between Curcumin and GO-Y030, and 2 ng/mL TGF-β versus all.

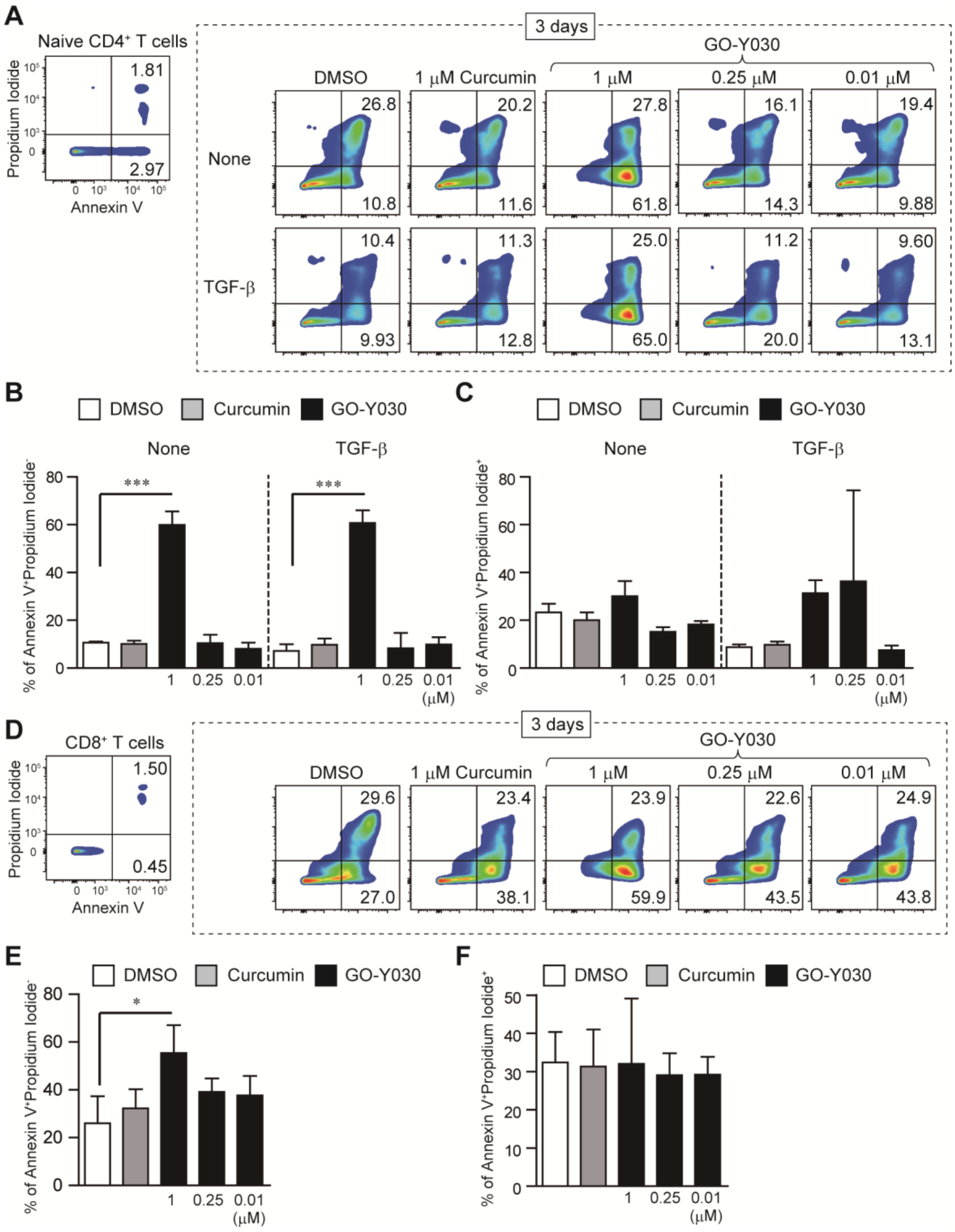

Supplementary Figure 2: Effects of GO-Y-030 in T cell viability.

(**A**) Representative Annexin V and Propidium Iodide FACS analyses at three independent experiments. Purified naïve CD4$^+$ T cells cultured for three days, and then gated CD4$^+$ cell population. (**B**, **C**) Statistic analyses were performed in each concentration between Curcumin and GO-Y030 versus DMSO control. One-way analysis of variance (ANOVA) with post-hoc Tukey's multiple comparisons test employed. (**D**) Representative Annexin V and Propidium Iodide FACS

analyses at three independent experiments. Purified naïve $CD8^+$ T cells cultured for three days, and then gated $CD8^+$ cell population. (**E**, **F**) Statistic analyses were performed in each concentration between Curcumin and GO-Y030 versus DMSO control. One-way ANOVA with post-hoc Tukey's multiple comparisons test employed.

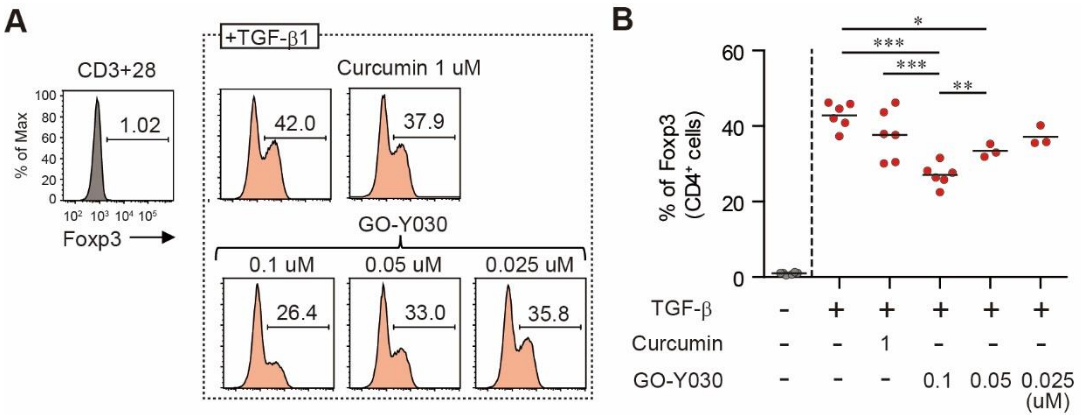

Supplementary Figure 3: GO-Y030 prevent TGF-β induced Foxp3$^+$Tregs generation

(**A**, **B**) Frequency of Foxp3$^+$ Tregs in total CD4$^+$ cells. Splenic naïve CD4$^+$T cells were cultured in the presence or absence of 2 ng/mL TGF-β, 1 μM curcumin, or 0.1-0.025 uM GO-Y030 for three days. One-way analysis of variance (ANOVA) with post-hoc Tukey's multiple comparisons test employed.

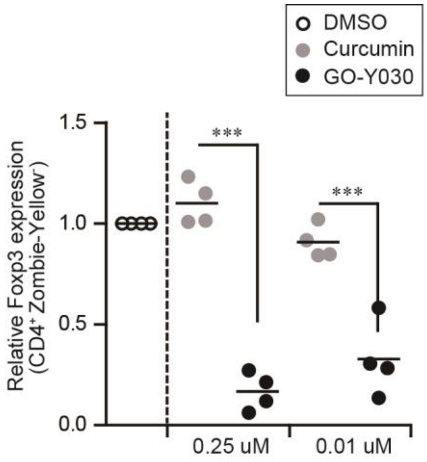

Supplementary Figure 4: Relative Foxp3$^+$Tregs in CD4$^+$Zombie Yellow$^-$ T cells.

Splenic naïve CD4$^+$ T cells were cultured in the presence of 2 ng/mL TGF-β with or without curcumin or GO-Y030 for three days. Percentage of TGF-β-induced Foxp3$^+$ population is as set as "1". One-way analysis of variance (ANOVA) with post-hoc Tukey's multiple comparisons test employed.

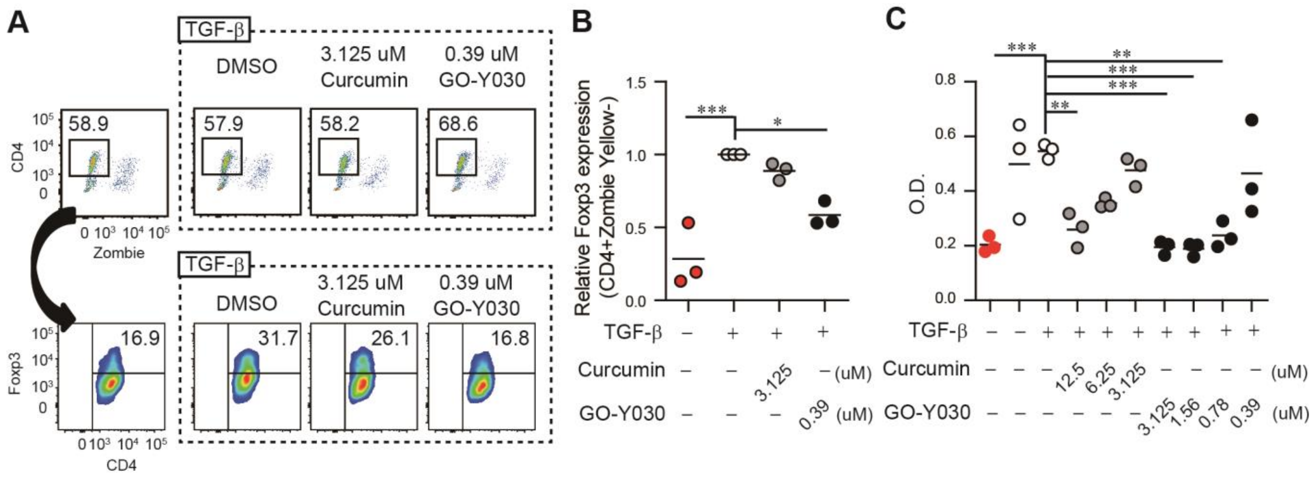

Supplementary Figure 5: GO-Y030 prevents TGF-β-induced Foxp3^{+}Tregs in human naïve CD4^{+} T cells.

(**A**) Human naïve CD4^{+}T cells were cultured in the presence of 2 ng/mL TGF-β1 with or without curcumin or GO-Y030 for three days. Data are one representative at three independent experiments. (**B**) Relative Foxp3 expression in CD4^{+} T cells. TGF-β1 stimulation only is set as "1". One-way analysis of variance (ANOVA) with post-hoc Dunnet's multiple (vs. TGF-β) comparisons test employed. (**C**) Relative live cell counts. Human naïve CD4^{+} T cells were cultured with or without 2 ng/mL TGFβ and concentrations of Curcumin or GO-Y030 as indicated for 72 h followed by the addition of the cell counting reagent. Red: No cells (Medium alone). Data are representative at three independent experiments using different healthy donors. One-way ANOVA with post-hoc Dunnet's multiple (vs. TGF-β) comparisons test employed.

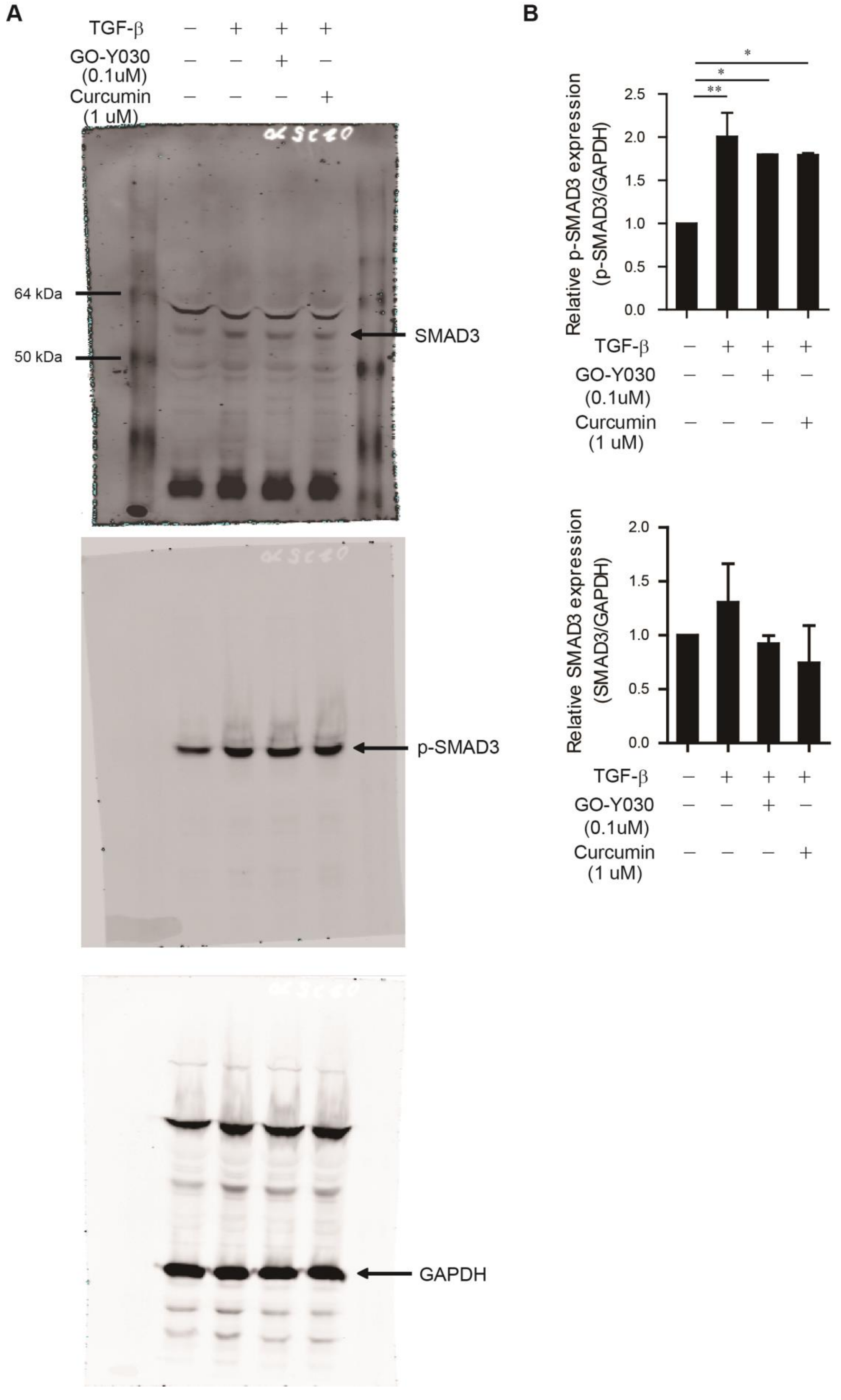
A
TGF-β – + + +
GO-Y030 (0.1uM) – – + –
Curcumin (1 uM) – – – +
64 kDa
50 kDa
SMAD3
p-SMAD3
GAPDH
B
Relative p-SMAD3 expression (p-SMAD3/GAPDH)
2.5
2.0
1.5
1.0
0.5
0.0
*
*
**
TGF-β – + + +
GO-Y030 (0.1uM) – – + –
Curcumin (1 uM) – – – +
Relative SMAD3 expression (SMAD3/GAPDH)
2.0
1.5
1.0
0.5
0.0
TGF-β – + + +
GO-Y030 (0.1uM) – – + –
Curcumin (1 uM) – – – +

Supplemental Figure 6: GO-Y030 does not affect TGF-β-induced SMAD pathway.

(**A**) Representative western blotting image of SMAD3, phospho-SMAD3 and GAPDH at three independent experiments. Naive $CD4^+$ T cells were stimulated with or without TGF-β in the presence or absence of 0.1 uM GO-Y030 or 1 uM Curcumin. (**B**) Relative SMAD3 and phospho-SMAD3 expression. (n=3, Mean with standard error of the mean) Without TGF-β and GO-Y030 stimulation was set as “1”. One-way analysis of variance (ANOVA) with post-hoc Tukey’s multiple comparisons test employed. One-way ANOVA with post-hoc Tukey’s multiple comparisons test employed.

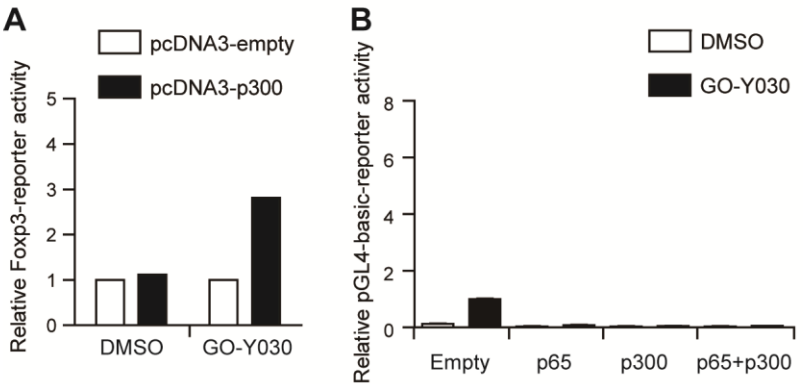

Supplemental Figure 7: Relative murine Foxp3-promoter activity

(**A**) pGL4-Foxp3 promoter (-1702 to +174) activity were analyzed using the Duo-luciferase assay systems. DMSO- or 1.0 uM GO-Y030 were treated HEK293 before 24 h electroporation. pcDNA3-empty vector transfection (Firefly/Renilla) was set as “1”. (**B**) pGL4-basic promoter (control) activity was analyzed using the Duoluciferase assay systems. DMSO- or 1.0 uM GO-Y030 were treated HEK293 cells before 24 h transfection. Data are one representative at two independent experiments (n=2, Mean + standard deviation).

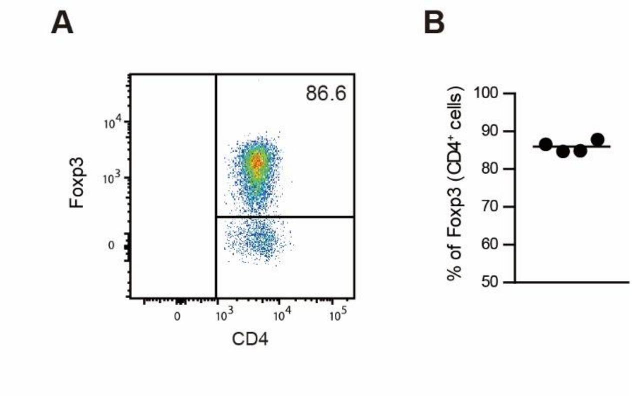

Supplemental Figure 8: Representative Foxp3 expression in Tregs after CD4$^+$CD25$^+$ isolation.

(**A**, **B**) Splenic CD4$^+$CD25$^+$ Tregs were isolated by using autoMACS (Milteny Biotech) and FACS analyses. Data are one representative of three independent experiments.

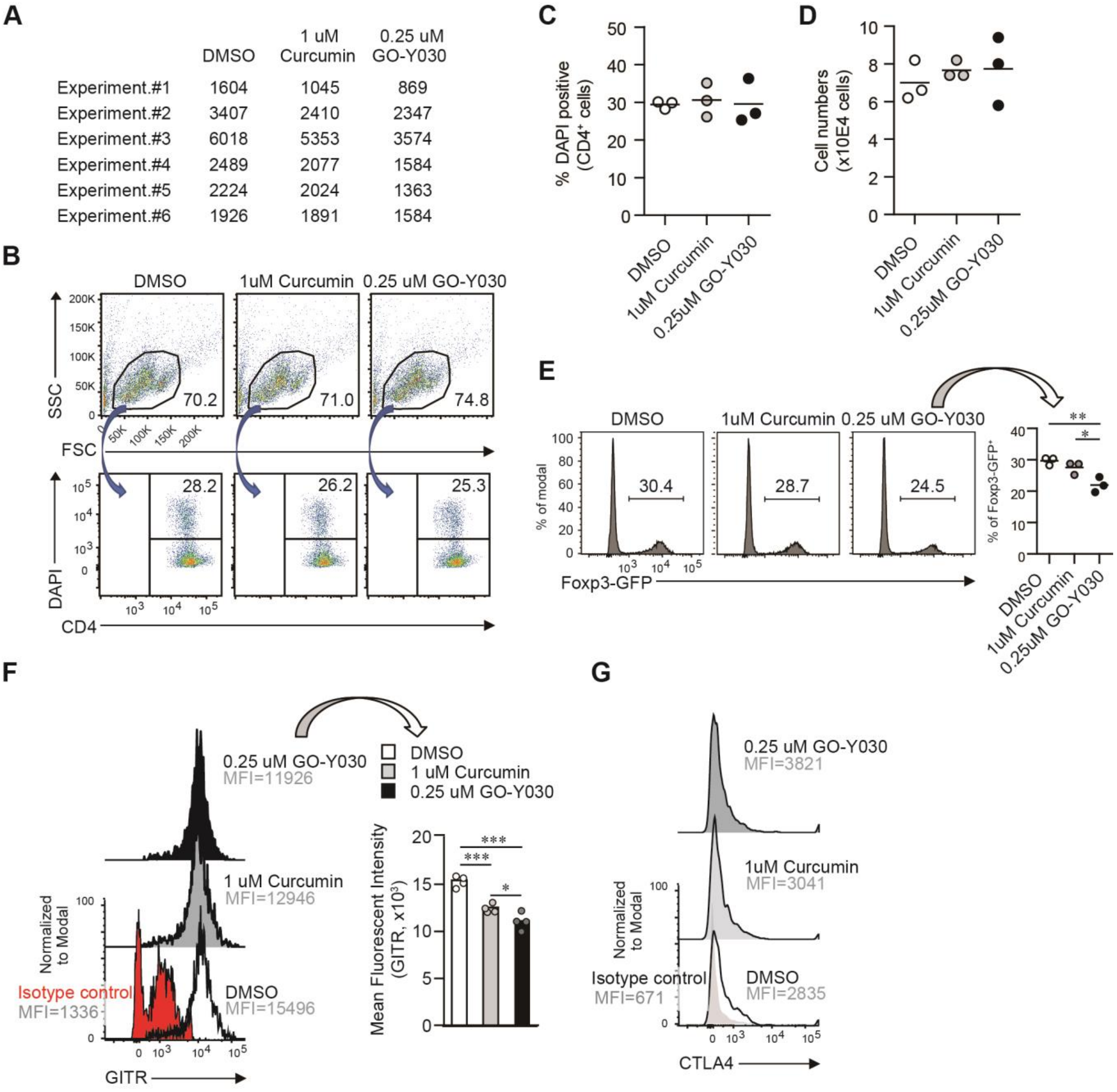

Supplemental Figure 9: Phenotype of Cultured CD4^{+}CD25^{+} Tregs.

(**A**) Foxp3 Mean fluorescence Intensity in CD4^{+}Foxp3^{+} Treg populations in Figure.3A. Data shows six independent experiments. experiments. (**B**, **C**) Relative cell survival rate after 18 h culture of CD4^{+}CD25^{+} Tregs with or without Curcumin or GO-Y030. Data are one representative at more than three independent experiments. (**D**) Absolute number of cells after 18 h culture of CD4^{+}CD25^{+} Tregs with or without Curcumin or GO-Y030. The starting number of cells in each wells was 1 x 10^{5} cells. (**E**) Foxp3-GFP positive cells in CD4^{+} population. Foxp3-GFP positive cells were purified by FACS-Aria II (>95%) and cultured 18 h with CD3 + CD28. Data are one representative at three independent experiments. GITR (**F**) and CTLA4 (**G**) in CD4^{+}Foxp3^{+} Treg populations in Figure. 5A. Gray; Isotype control, Black; GITR or CTLA4. Data are one representative at three independent experiments. One-way analysis of variance with Tukey employed for statistic difference.

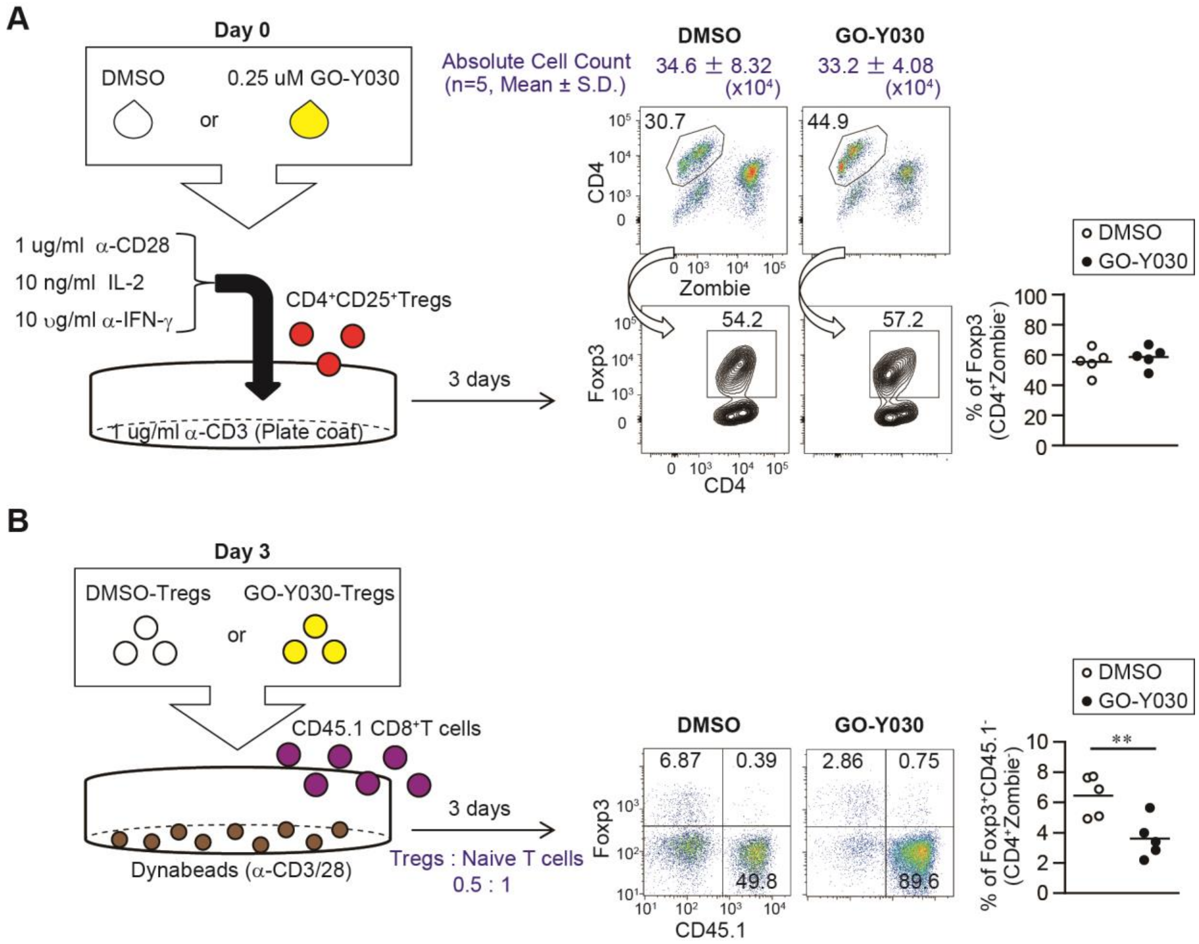

Supplemental Figure 10: GO-Y030 controls IL-2/STAT5 axis in CD4$^+$CD25$^+$ Tregs.

(**A**) Percentage of Foxp3 in cultured CD4$^+$CD25$^+$Tregs at day three. Data are representative at five independent experiments. Student T-test was employed. (**B**) Percentage of Foxp3 in cultured CD4$^+$CD25$^+$ Tregs in the suppression assay. DMSO- or GO-Y030-treated CD4$^+$CD25$^+$ Tregs were co-cultured with CD8$^+$ T cells (Tregs:CD8$^+$ T cells=0.5:1) for three days. Data are representative at shows five independent experiments. Student T-test was employed.

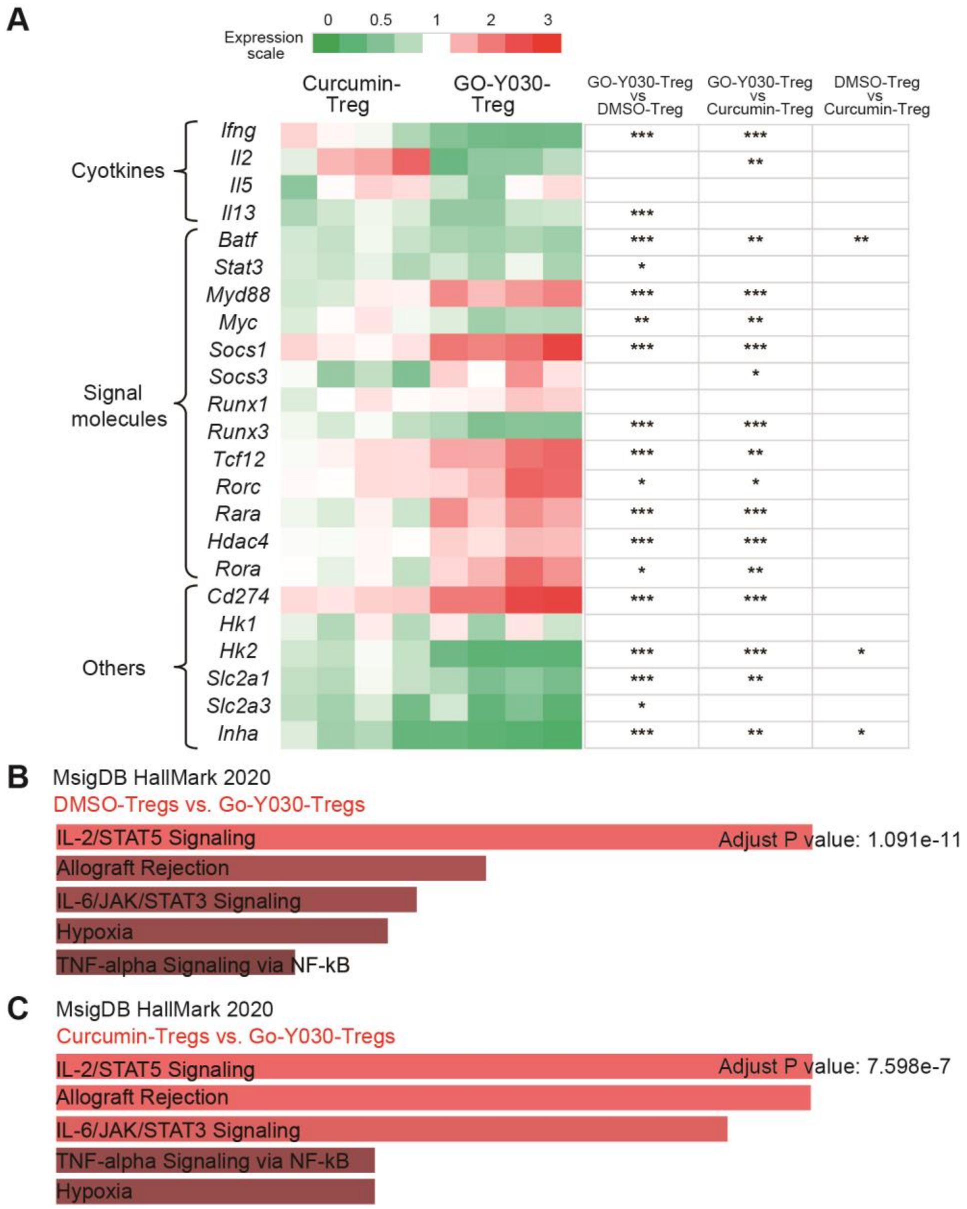

Supplemental Figure 11: GO-Y030 controls IL-2/STAT5 axis in CD4^{+}CD25^{+} Tregs.

(**A**) Real time PCRs in 72 h culture of CD4^{+}CD25^{+} Tregs with or without Curcumin or GO-Y030. The color scale is shown at the top of heat map. Each genes expression of DMSO-treated Tregs are as set as "1". Data showed four independent experiments. (**B, C**) Enrichr- was used to calculate enrichment scores of signaling pathways. We selected genes of significantly difference expression (*P<0.05*) between DMSO-Tregs and GO-Y030-Tregs (**B,** https://maayanlab.cloud/Enrichr/enrich?dataset=ce1ae4783a07360aa829bddb0fd36eb1) or Curcumin-Tregs and GO-Y030-Tregs (**C,** https://maayanlab.cloud/Enrichr/enrich?dataset=35a8b176b017fbdc7c09576aa75995cd). Statistical analyses (One-way analysis of variance with Tukey) were performed.

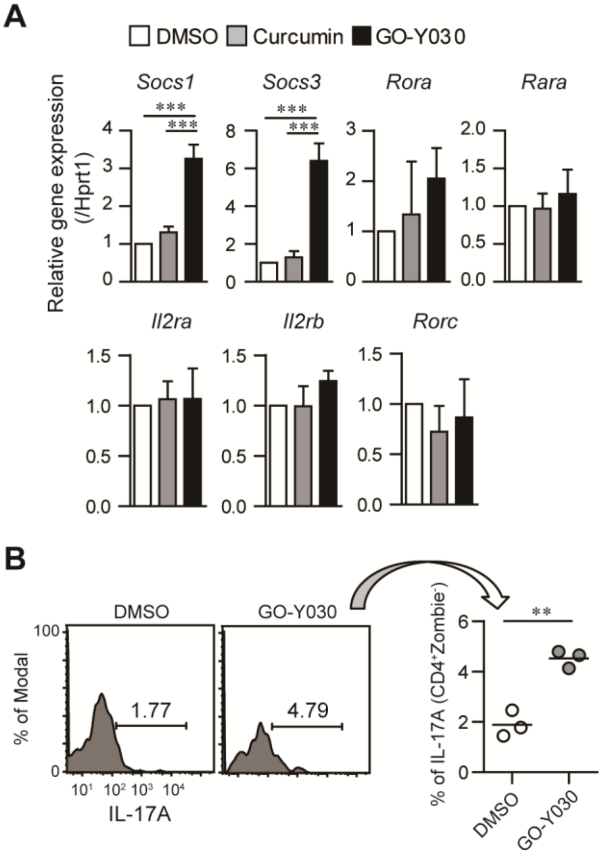

Supplemental Figure 12: GO-Y030 controls IL-2/STAT5 axis in CD4$^+$Foxp3-GFP$^+$ Tregs.

(**A**) Real time PCRs in 72 h culture of CD4$^+$Foxp3-GFP$^+$ Tregs with or without 1 μm Curcumin or 0.25 μm GO-Y030. Data pooled three independent experiments. Statistical analyses (One-way analysis of variance with Tukey) were performed. (**B**). Th17 population in cultured CD4$^+$Foxp3-GFP$^+$ Tregs with or without 0.25 μm GO-Y030. Student T-test was performed.

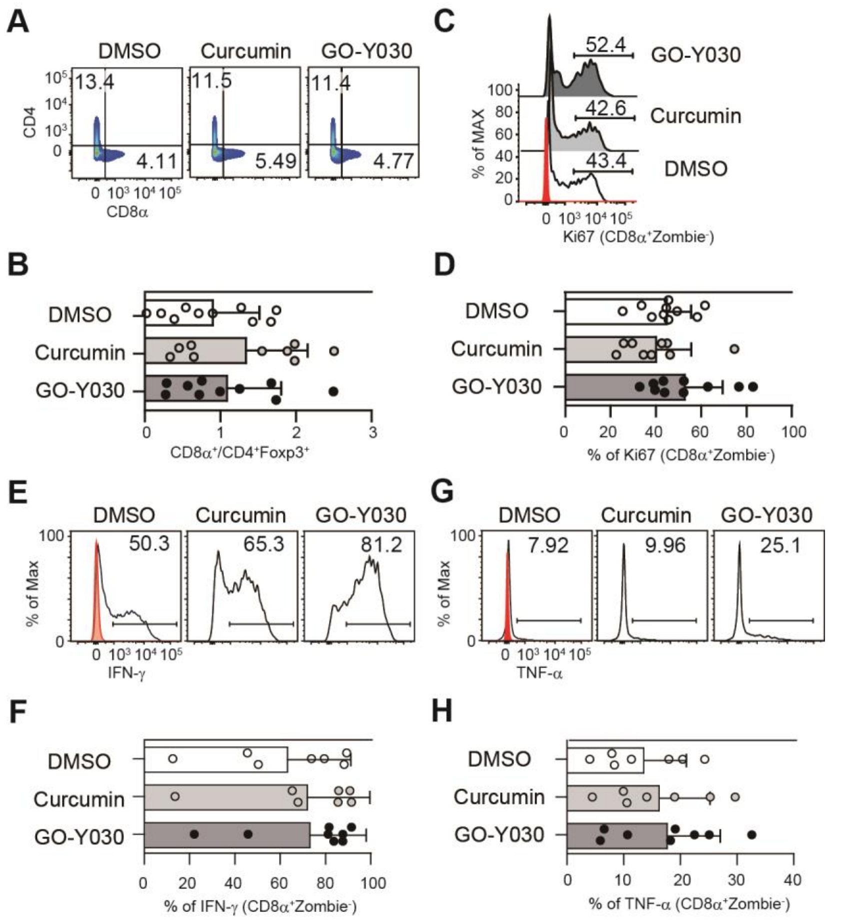

Supplemental Figure 13: GO-Y030 did not prevent infiltration and activation of CD8$^+$ cells in tumor microenvironment.

(**A**) Frequency of CD4$^+$/CD8α$^+$ cells in tumor infiltrate lymphocytes. (**B**) Ratio of tumor infiltrate CD8$^+$ cells to CD4$^+$Foxp3$^+$ Treg cells. (**C**, **D**) Ki67 expression in CD8α$^+$ cells in tumor infiltrate lymphocytes. Red; isotype control. Data are one representative of each of the two independent experiments (**A**, **C**). (**E**, **F**) IFN-γ production from CD8α$^+$ cells in tumor infiltrate lymphocytes. Red; isotype control. (**G**, **H**) TNF-α production from CD8α$^+$ cells in tumor infiltrate lymphocytes. Red; isotype control. One-way analysis of variance with post-hoc Tukey's multiple comparisons test was used (**B**, **D**, **F**, **H**). The graph shows mean and standard deviation.
